# Broad-Spectrum Antimicrobial, Antioxidant, and Anticancer Studies of Leaf Extract of *Simarouba glauca* DC In Vitro

**DOI:** 10.3390/antibiotics11010059

**Published:** 2022-01-03

**Authors:** Shanmuga Priya Ramasamy, Anitha Rajendran, Muthukrishnan Pallikondaperumal, Priya Sundararajan, Fohad Mabood Husain, Altaf Khan, Mohammed Jamal Hakeem, Abdullah A. Alyousef, Thamer Albalawi, Pravej Alam, Hazim M. Ali, Abdulaziz Alqasim

**Affiliations:** 1 Department of Microbiology, PSG College of Arts & Science, Coimbatore 641014, India; anitha87blemish@gmail.com (A.R.); pmamatricschool@gmail.com (M.P.); priyasundararajan2509@gmail.com (P.S.); 2 Department of Food Science and Nutrition, King Saud University, Riyadh 11451, Saudi Arabia; mhakeem@ksu.edu.sa; 3 Central Laboratory, Department of Pharmacology and Toxicology, College of Pharmacy, King Saud University, Riyadh 11451, Saudi Arabia; altkhan@ksu.edu.sa; 4 Clinical Laboratory Science Department, College of Applied Medical Sciences, King Saud University, Riyadh 11451, Saudi Arabia; abalyousef@ksu.edu.sa (A.A.A.); aalqasim@ksu.edu.sa (A.A.); 5 Department of Biology, College of Science and Humanities, Prince Sattam Bin Abdulaziz University, Alkharj 11942, Saudi Arabia; t.albalawi@psau.edu.sa (T.A.); alamprez@gmail.com (P.A.); 6 Department of Chemistry, College of Science, Jouf University, P.O. Box 2014, Sakaka 72388, Saudi Arabia; hmali@ju.edu.sa

**Keywords:** *Simarouba glauca*, terpenoids, antimicrobial, antioxidants, anticancer, MCF-7 cell line

## Abstract

The current study aimed to screen the preliminary phytochemicals in the leaf extract of the medicinal plant *Simarouba glauca* and to analyze its potential antimicrobial, antioxidant and anticancer properties. The phytochemical profile of the methanol extract was analyzed, and bioactive compounds were identified using chromatography, FTIR and GCMS. Antimicrobial activity and Minimum Inhibitory Concentration (MIC) were determined against 14 bacterial and 6 fungal strains. Moreover, the synergistic effect of a plant extract with commercially available antibiotics was also evaluated using the checkerboard method. The ethanolic and methanolic extracts showed exclusive activity against *S. aureus* and profound activity against *E. coli* and *S. marcescens*. Upon comparing breakpoints, methanolic extract demonstrated higher antimicrobial activity with a MIC value of 3.2 mg/mL against the test pathogens. Furthermore, the extracts demonstrated potential antioxidant activity; methanol extract had higher antioxidant potential compared to the ethanol extract. The major proactive bioactive compound with maximum antioxidant capacity was observed to be terpenoids. The methanol extract of *S. glauca* showed significant cytotoxicity against the MCF-7 breast cancer cell line with an IC_50_ value of 16.12 µg/mL. The overall results of our work provide significant evidence for the usage of methanolic extract of *S. glauca* as an efficient ethnomedicinal agent and a potential candidate for relieving many human ailments.

## 1. Introduction

Cancer is one of the major causes of death globally, accounting for approximately 9.6 million deaths in 2018. It is estimated that death could be increased by around 13 million by 2030 [1]. Among various types of cancers, breast cancers are commonly found due to lack of awareness and negligence to preliminary symptoms. Breast cancer is considered a leading cause of death worldwide. The mortality of breast cancer is mainly due to the metastasis of primary cancer to different sites, including organs such as the bones, brain, liver, lymph nodes and lungs. Moreover, an increased drug resistance pattern towards existing antibiotics by pathogens is a serious threat. The drug-resistant phenotypes further increase the risk of cancer prevalence and render the current therapies ineffective [2,3]. The existing treatment for breast cancer is complex and is associated with toxic side effects. The 5-year survival rate of metastatic breast cancer patients is approximately 25%, suggesting the importance of targeted therapy for metastasis. In a few cases, chemotherapy or radiotherapy may also be applied after surgery to reduce the severity of the infection. However, due to the deleterious side effects caused in human beings, a search for novel candidates with multifarious activity is required. The presence of antimicrobial compounds and natural antioxidants in plants that remove harmful free radicals is one of the major reasons for using plant extracts as a potential tool for treating drug-resistant pathogens and cancer. Medicinal implications of plants are well known to prevent and minimize the harmful effects of conventional treatments. Moreover, combinatorial therapy provides a promising view in using the plant extract against antibiotic-resistant microbes and dreadful diseases such as cancer [4,5,6,7].

*Simarouba glauca,* commonly known as Laxmitaru or the Paradise tree and grouped under the family *Simaroubaceae,* is distributed widely in tropical regions. This plant has been traditionally used in the treatment of various diseases, as it possesses multi-medicinal properties [1]. The *S. glauca* plant extract is known to possess a bioactive compound that has significant therapeutic applications. Quassinoids are one of the major active phytochemical constituents that belong to the triterpene family, exhibiting significant pharmacological properties such as antimicrobial, anticancer, antipyretic and haemostatic activity. These compounds inhibit nucleic acid and protein synthesis via interference at the peptidyl transferase site or through the downregulation of phosphoribosyl pyrophosphate aminotransferase, which confers anticancer activity [8,9]. In general, different parts of *S. glauca* has been used in the treatment of wide range of diseases. However, only limited studies have been reported towards the broad-spectrum antibacterial, antifungal and anticancer efficacy of *S. glauca* extract against breast cancer.

Hence, the present study aimed to evaluate the antimicrobial and anticancer activity of the methanolic extract of *S. glauca* on MCF-7 breast cancer cell lines. Antimicrobial activity was assessed against 14 bacterial and 6 fungal strains. Furthermore, phytochemical characterization was performed to detect the presence of bioactives such as alkaloids, flavonoids, phenolic compounds, terpenoids, tannins, glycosides etc., that play a crucial role in the biological activity of the extract. In addition, the synergistic and combinatorial effects of the plant extract were used to determine the efficacy of *S. glauca*. The anticancer potential of the extract was also evaluated against the MCF-7 breast cancer cell lines.

## 2. Results and Discussion

### 2.1. Phytochemical Screening of Plant Extract

The ethanol and methanol extract of *S. glauca* was obtained through the Soxhlet apparatus. The dried powder was collected and stored in airtight bottles at 4 °C until further analysis. Both ethanol and methanol extracts of *S. glauca* subjected to phytochemical screening showed the presence of predominant phytochemicals. The results have been tabulated (Table 1). The results revealed the presence of alkaloids, flavonoids, phenols, steroids, terpenoids, tannins, glycosides, saponins, carbohydrates and fixed oils. Similar results were reported for the presence of phytochemicals in methanol extract of *S. glauca* by Pawar et al. [9] and Lakshmi et al. [10].

### 2.2. Antimicrobial Activity of Plant Extract

The antibacterial and antifungal activity of the ethanol and methanol extract of *S. glauca* was evaluated, and the results showing its zone of inhibition were tabulated (Table 2). It had been noted that the extract of *S. glauca* exhibited varying degrees of antibacterial and antifungal activity against all of the bacterial and fungal strains tested. The highest zone of inhibition was recorded against *S. aureus*, *E. coli* and *S. marscescens*. The extract was found effective in inhibiting the growth of the test fungal strains, and zone size ranged from 12.5–18 mm. The above results were in accordance with the results of Lakshmi et al. [10] and Karthikeyan et al. [11]. The bacteria showed more susceptibility than fungi which may be due to the presence of lipid layers, extra lipopolysaccharides. The cell wall matrix of the bacteria could possibly be solubilized by polar substances such as terpenoids, steroids, essential oils, etc., which are the main active plant chemicals and are responsible for the waxy coat of the leaves preventing microbial attack [12].

### 2.3. Minimum Inhibitory Concentration (MIC)

Comparing the methanol, ethanol extract and streptomycin sulphate, the methanol extract exhibits higher antibacterial activity than the ethanol extract (Table 3). However, the streptomycin sulphate was on par even in the microgram concentration. From the results obtained, *S. aureus* was highly susceptible to methanol extract, and the MIC value was recorded as 3.2 mg/mL. This was in agreement with the results of Jangale et al. [13], where the methanol extracts of the *S. glauca* leaves exhibited a MIC of 640 ppm against *S. aureus*. The antimicrobial activity is higher in methanol extracts when compared with ethanol extracts. This may be because some active compounds being polar, readily dissolve in methanol which has higher polarity than ethanol [14]. From the well diffusion assay and the MIC, it was noticed that *S. aureus* was the most susceptible clinical pathogen, and hence it was selected for further study.

### 2.4. Mode of Action of Methanolic Extract of S. glauca towards Staphylococcus aureus

**Figure 1:**
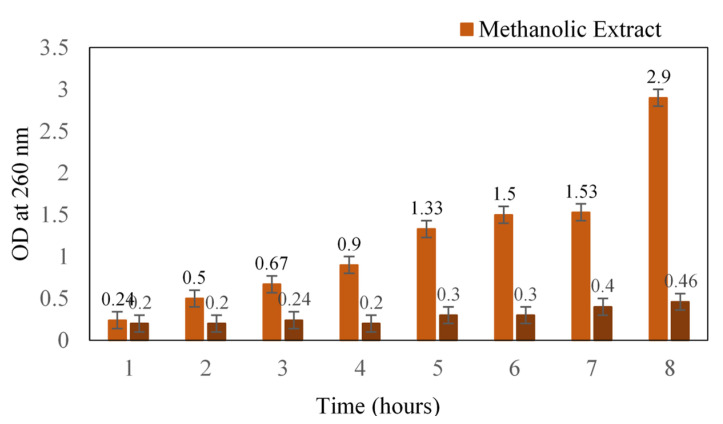
Loss of cellular content upon treatment with methanol extract of *Simarouba glauca* against *S. aureus.* Absorbance was read at 260 nm.

The mode of action of methanolic extract was determined in terms of leakage of cellular content. Initially, there was a slight increase in the absorbance that might be due to the starting of leakage, after which a reasonable increase was noted (Figure 1). At the 12th hour, the absorbance was 1.5, and after 24 h, absorbance was recorded as 2.9. A similar antibacterial study of black pepper extract on *S. aureus* reported the loss of cellular material at 260 and 280 nm [15]. The methanol extract of *S. glauca* might have caused significant membrane damage to the cell wall of the cocci, which would have resulted in the leakage of cytoplasmic materials such as nucleic acids. Moreover, our plant extract is known to possess quassinoids, a terpenoid that could plausibly be one of the main reasons for its antibacterial action [16].

### 2.5. Scanning Electron Microscopy

**Figure 2:**
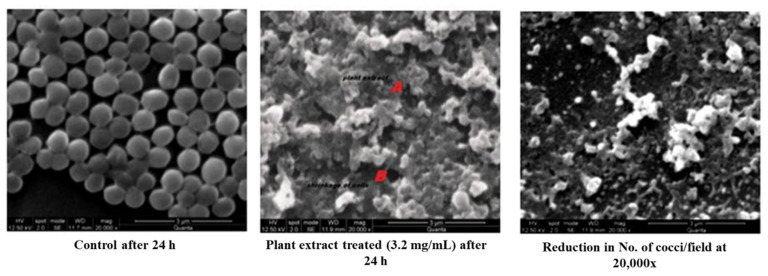
SEM analysis of control and plant extract treated *S. aureus*.

A remarkable morphological difference was observed in the plant extract (MIC of 3.2 mg/mL) treated *Staphylococci* than that of the control (Figure 2). An SE micrograph of the untreated control clearly shows the spherical structure of cocci, whereas significant cell shrinking and distorted cell structures can be observed in the plant extract-treated cells. It was also observed that the number of cocci per field was profoundly reduced when compared with the control, which indicated the cell death and the cellular debris of the disrupted cell could also be visualized. The extract would have solubilized the lipid contents of the cell wall and extensively damaged the cellular matrix resulting in leakage of intracellular components with a massive shrinkage of cells. A significant structural loss was almost seen in the cocci treated with plant extract. Thus, the severity of the methanolic extract of *S. glauca* DC., against *S. aureus* has been proven by SEM analysis. Similarly, aqueous extracts of *Rhazya stricta* were treated against *S. aureus,* and prominent cell leakage was observed at each stage where the cell lost its shape with maximum leakage of the cytoplasmic materials [17].

### 2.6. Estimation of Protein Content

**Figure 3:**
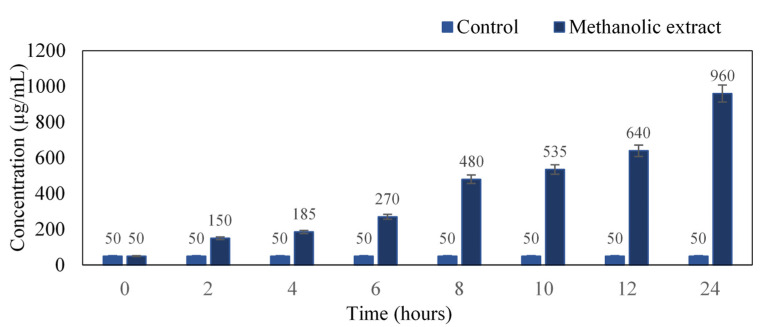
Quantification of protein in the cell culture from 0–24 h.

The intensity of the color developed is directly proportional to the concentration of proteins present in the culture medium. There was a profound increase in protein concentration with respect to time. At the 24th hour, the concentration was found to be 960 µg/mL as compared to the untreated control (Figure 3). Our results coincide with Sathyabama et al. [18], where *S. aureus* when treated with triterpenoids compound of *Tridax procumbens*, resulted in protein leakage of 60.12 µg/mg at 18 h of incubation.

### 2.7. Isolation and Analysis of Bacterial Proteins by SDS PAGE

**Figure 4:**
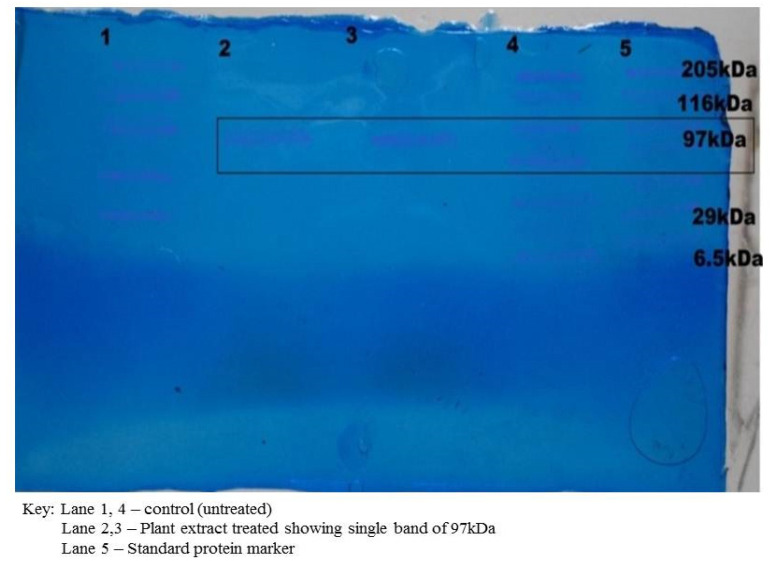
Protein analysis by SDS PAGE.

The dialyzed cell protein sample was subjected to SDS-PAGE. It was observed that the protein sample treated with the plant extract varied from the control (untreated). One band of 97 kDa of molecular weight was observed in the treated protein sample, which showed that the plant extract had lysed and disrupted the cell protein of the *S. aureus* (Figure 4). It had earlier been reported that polypeptide profiles of the multiple drug-resistant clinical strain *S. aureus*, when exposed to the extracts of the leaf of *Pisidium guajava*, exhibited a strong proteolytic activity with a band of 29 kDa [19].

### 2.8. Synergism Test by Checkerboard Method

From the results of the checkerboard analysis (Table 4), it was noted that among the three different concentration ranges (1/2 MIC, MIC and 2× MIC) of a plant extract with the antibiotic streptomycin, the MIC and 2× MIC concentration exhibited synergism possessing fractional inhibitory concentrations of 0.5 and 0.375. The combination of streptomycin with methanol extract of *S. glauca* can effectively exhibit synergistic activity against *S. aureus*. However, the molecular basis of synergistic interactions and mechanisms which are fundamental to developing pharmacological agents to treat bacterial infections has to be studied. Similar results were reported by Chovanova et al. [20], where a synergism of crude extract of *Salvia officinalis* along with the antibiotic oxacillin against *S. epidermidis,* was observed and reported to have an FIC of 0.14.

### 2.9. Isolation and Identification of Bioactive Compound through Column Chromatography and Its Antibacterial Assay

Among the four fractions tested, fraction 3, which was brown in color, showed higher activity (Table 5), and hence that fraction alone was taken for further identification and characterization.

#### 2.9.1. FT-IR Analysis of Column Fraction

The functional groups of the F3 column fraction of *S. glauca* extract were characterized, and the results were tabulated (Table 6). The major peaks of absorption and the respective functional groups were identified. According to Starlin et al. [21], the absorption peak at 1705.07–1720.8 cm^−1^ represents C=O stretching as a ketone group which may be due to the presence of terpenoid compounds. Our extract also exhibits similar vibrations at the desired region, which evidenced that the test compound may have terpenoids.

#### 2.9.2. Gas Chromatography–Mass Spectroscopy

The active phytochemical constituents present in the methanolic extract of *S. glauca* (Fraction 3) were determined. Approximately eight compounds were identified based on their retention time (RT), molecular formula, molecular weight (MW) and concentration (%). This was compared with the NIST and WILEY database, and the results are tabulated (Table 7). The terpenoid class of compound are the major phytochemicals present in the *S. glauca*, and these are found to be responsible for pharmacological actions, including antibacterial activity, which is evident in our study.

#### 2.9.3. Analytical TLC of Column Fraction

**Figure 5:**
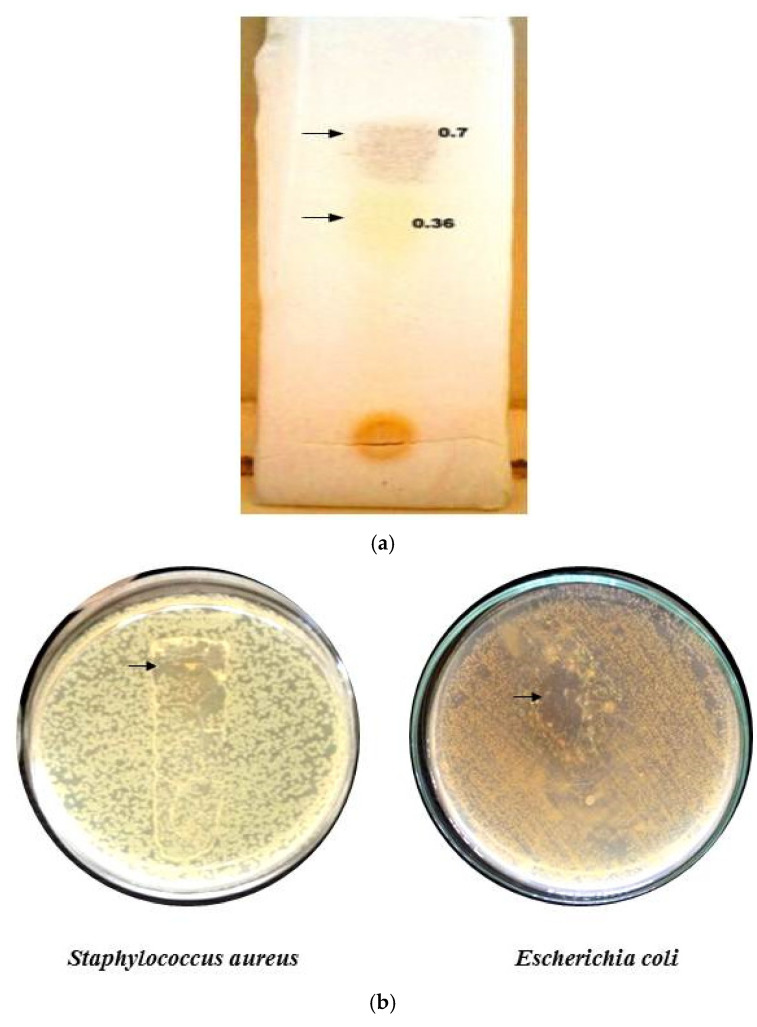
TLC of F3 column fraction (**a**) and Bioautography (**b**).

The Rf values of column fraction 3 were determined through TLC. Two visible spots were determined. A brown color spot and a yellow spot have Rf values of 0.7 and 0.36, respectively (Figure 5a). Biradar and Rachetti [22] reported a similar Rf value when the triterpenoid extract of *Centella asiatica* leaf was analyzed through TLC. Thus, from the Rf value of the fraction obtained, it was evident that the fraction contained a terpenoid class of compounds in higher concentrations.

#### 2.9.4. TLC Bioautography

A clear zone at the contact point of the separated spots in the plate inoculated with *S. aureus* and *E. coli* was observed (Figure 5b). The result indicates that the separated compound possessed the bioactive property, as reported earlier by Rajiniraja and Jayaraman [23]. Thus, from the above tests, it was clear that the identified fraction 3 belonged to the terpenoids class and possessed the bioactive property. However, further purification, structural elucidation and characterization need to be conducted for the compound to act as the potential source of the therapeutic drug.

### 2.10. Antioxidant Activity of Simarouba glauca Extract

**Figure 6:**
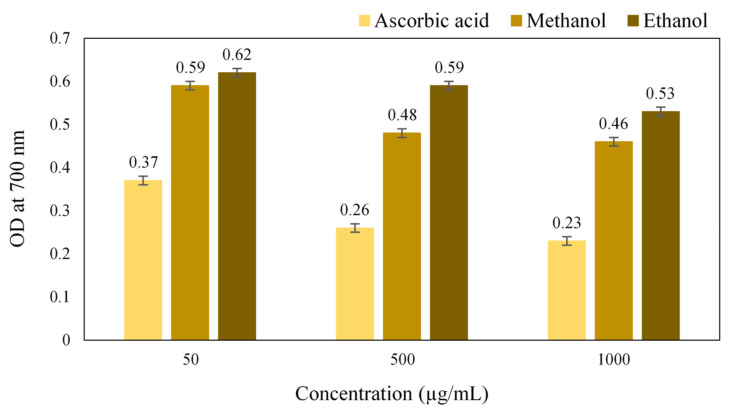
Antioxidant activity of *Simarouba glauca* extracts (Reducing power activity).

A gradual increase in the reducing power with an increase in the concentration of the extract was obtained, which was due to the presence of plant chemicals that reduces the Ferric (Fe^3+^) ions to Ferrous (Fe^2+^) ions. The results of ferric reducing activity (Figure 6) showed that the methanol extract has higher antioxidant potential compared to the ethanol extract. The reducing power is an indication of the presence of a reducing agent having the availability of atoms that can donate electrons to react with free radicals and then convert them into more stable metabolites and terminate the radical chain reaction [24]. Thus, it revealed that the crude extract of *S. glauca* had the reducing agents that can scavenge the free radicals.

### 2.11. Anticancer Activity of S. glauca Extract 

**Figure 7:**
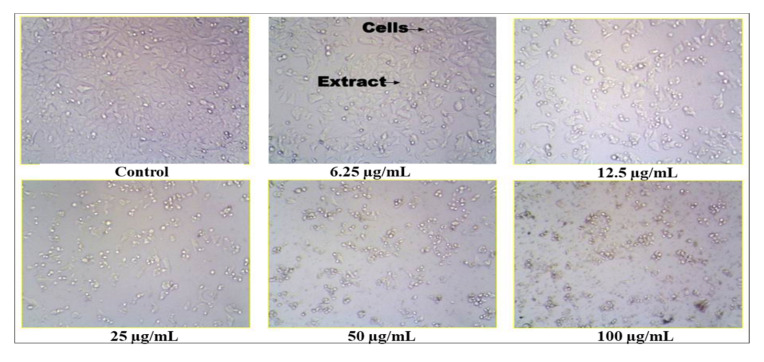
Anticancer activity of *Simarouba glauca* extract on MCF-7 cell line.

The crude methanol extract of *S. glauca* was found to be inhibitory to the MCF 7 cell lines. The values are tabulated (Table 8, Figure 7), and it was observed that when the concentration of the plant extract was increased, the cell inhibition percentage also increased correspondingly. Thus, the concentration of the extract and the cell inhibition is directly proportional, and the cytotoxic activity was found to be dose-dependent. In addition, the IC_50_ (half maximal inhibitory concentration) was found to be 16.12 μg/mL. Similarly, in an earlier study, a new quassinoid, designated 2′-(R)-O-acetylglaucarubinone of *Quassia gabonensis* Pierre of *Simaroubaceae* family, recorded an IC_50_ of 8.13 µg/mL against MCF-7 breast cancer cell lines [25]. Suffness et al. [26] reported that cytotoxicity activity for the crude extracts, as established by the American National Cancer Institute (NCI), must have a (half maximal inhibitory concentration), i.e., IC_50_ ˂ 30 µg/mL in the preliminary assay. Our plant extract fulfills this criterion which helps us proceed further in the in vivo study. Thus, from the antioxidant and cytotoxic study, it was noticed that the extract contains compounds that are capable of scavenging the free radicals, thereby preventing cancer incidence.

## 3. Materials and Methods

### 3.1. Collection and Preparation of Plant Extracts

The fresh, disease-free leaves of *S. glauca,* approximately 1 kg, were collected and washed thoroughly 2–3 times with tap water as well as autoclaved distilled water. Later, the leaves were shade dried at room temperature for a week. The dried leaves were then crushed to a fine powder and stored in airtight bottles until further use. Roughly 5 g of dried leaf powder was mixed in 100 mL of each methanol and 99% ethanol, respectively. The content was packed in a Soxhlet apparatus and was subjected to Soxhlet extraction for 3 h, 3 cycles at 30 °C. The plant extracts were then filtered using Whatman No: 1 filter paper, the filtrate was subjected to solvent evaporation at 30 °C and dry powder extract was obtained. The powder extract was preserved in airtight bottles at 4 °C until further use [10].

### 3.2. Phytochemical Screening

The phytochemical screening for methanol and ethanol extract of *S. glauca* were tested for the presence of phytochemicals, namely, alkaloids, flavonoids, carbohydrates, phenolic compounds, tannins, triterpenoids, saponins, steroids and glycosides by the standard procedures as described in the literature [27,28].

### 3.3. Antimicrobial Activity of Plant Extract

#### 3.3.1. Screening of Antibacterial Activity

The antibacterial activity was screened using an agar well diffusion assay. The Muller–Hinton agar (MHA) medium was prepared and poured into the sterile Petri plate, and allowed to solidify. The 100 µL of 0.5 McFarland standardized bacterial inoculum was swabbed on an MHA medium. Then the wells of 9 mm in diameter were made in the medium using a sterile cork borer. A total of 100 µL of ethanol and methanol extracts were transferred into separate wells, and the plates were incubated at 37 °C for 24 h. The streptomycin (100 µg/mL) served as a positive control. Followed by incubation, the plates were observed for the presence of a clear zone of inhibition around the well, indicating antibacterial activity, and zone size was measured in mm [11,14].

#### 3.3.2. Screening of Antifungal Activity

The antifungal activity was screened using an agar well diffusion assay. The Potato dextrose agar (PDA) medium was prepared and poured into the sterile Petri plate, and allowed to solidify. The 100 µL of 0.5 McFarland standardized fungal inoculum was swabbed on a PDA medium. Then wells of 9 mm in diameter were made in the medium using a sterile cork borer. A total of 100 µL of ethanol and methanol extracts were transferred into separate wells. The plates were incubated at room temperature for 5–6 days. The fluconazole (50 µg/mL) served as a positive control. Followed by incubation, the plates were observed for the presence of a clear zone of inhibition [29].

#### 3.3.3. Minimum Inhibitory Concentration (MIC)

The MIC was performed using a double broth macro dilution assay as per the NCCLS broth dilution procedure [30]. Briefly, approximately 0.2 g of *S. glauca* extract was dissolved in MHA, respectively, to give a final concentration of 100 mg/mL. They are serially diluted to attain a concentration of 50, 25, 12.5, 6.3, 3.2, 1.6, 0.8, 0.4, 0.2 mg/mL. Approximately 20 µL of 0.5 McFarland standardized bacterial cultures were aseptically inoculated and incubated at 37 °C for 24 h. Streptomycin served as a positive control.

### 3.4. Mode of Action of Methanolic Plant Extract on Staphylococcus aureus

The most sensitive organism among the tested clinical pathogens was identified and was taken for further study.

#### 3.4.1. Test for Loss of Cellular Content at 260 nm

The overnight culture of the *S. aureus* was centrifuged at 10,000 rpm for 15 min. The cell pellets were collected and washed twice with a normal saline solution. It was then re-suspended in the saline solution, and the final cell density was adjusted to 1.5 × 10^8^ CFU/mL at 600 nm by 0.5 McFarland’s turbidity method. The extract was added at a final concentration equivalent to the Minimum Inhibitory Concentration (MIC) to the suspension and was incubated in a shaker incubator at 37 °C for 24 h. The cell suspension without the plant extract was used as a control. At every 2 h, a 1 mL sample was withdrawn and centrifuged at 10,000 rpm for 10 min. Then the supernatant was collected, and the absorbance at 260 nm was measured against the saline solution as blank [31].

#### 3.4.2. Scanning Electron Microscopy (SEM)

To determine the effect of the plant extract on *S. aureus*, the treated plant extract (MIC) and the untreated cell culture were analyzed by scanning electron microscope. The bacterial culture was treated with the plant extract at its MIC, and the cells were recovered after 24 h by centrifugation at 10,000 rpm for 10 min. Then the cell pellet was washed twice with saline solution. The cells were then smeared as a thin film on the oil-free glass slide and were fixed using 5% glutaraldehyde for half an hour and then dehydrated with 70% ethanol for 15 min. The dehydrated samples were further dried under the blower to ensure complete drying. The sample was placed on double-sided carbon tape which was mounted on aluminum metal stubs and then sputter-coated with gold. The images were taken using an FEI Quanta 200 scanning electron microscope at 20,000× magnification with 12.5 kV as accelerating voltage [32].

#### 3.4.3. Estimation of Protein Content

The protein content was measured using the dye-binding method of Bradford, where Bovine Serum Albumin (BSA) was used as standard. The absorbance was read at 595 nm against 0.1 M phosphate buffer (pH 7.2) as blank. By using the optical density values, the standard curve was plotted. The culture filtrate was estimated for the protein concentration that may be due to the antibacterial effect of the plant extract, resulting in the leakage of cell content because of the membrane damage of the bacterial cell wall. Overnight broth culture of *S. aureus* was treated with MIC of the plant extract and was incubated in a shaker incubator at 37 °C for 24 h. The sample was recovered at every 2 h of the time interval and was centrifuged at 10,000 rpm for 10 min. Approximately 1 mL of supernatant (sample) was taken, and the Bradford reagent was added and allowed to stand for 5–10 min where the blue color was developed. The color developed as a result of dye binding to the protein present in the supernatant was directly proportional to the amount of protein present in the supernatant. The absorbance was read at 595 nm against the nutrient broth as blank. The protein concentration was quantified by the standard curve [19].

### 3.5. Isolation of Bacterial Proteins for SDS PAGE

The test bacterium (*Staphylococcus aureus*) was treated with the MIC of the extract and was incubated in a shaker for 37 °C for 24 h. The culture was centrifuged at 10,000 rpm for 10 min, and the supernatant and pellet were separated for precipitation. Approximately 40% of Ammonium sulphate was prepared, and approximately 1 mL was added to the cell pellets whereas, the supernatant was taken as a cytoplasmic fraction which was precipitated by adding 1.22 g/5 mL of the supernatant and was left undisturbed for precipitation. The precipitated pellet was centrifuged at 10,000 rpm for 10 min. The pellet containing ammonium sulphate was dissolved in 50 mM Tris-HCL of pH 8 and was subjected to dialysis. Pre-treatment of the dialysis membrane plays an essential role in dialysis. The dialysis membrane was cut to the desired length and was treated with double distilled water to remove glycol at 65 °C for 10 min. The membrane was then soaked in 10 mM EDTA containing a half pellet of NaOH. The membrane was then treated with 2% NaCO_3_ to remove sulphur molecules and washed with distilled water. The membrane was closed at one end by dialysis closure clips, and the bag was filled with the sample (pellet of treated and untreated). The other end was then closed with the closure clips. The bag was immersed in Tris HCL buffer of pH 8 and left undisturbed at 4 °C [33].

### 3.6. SDS-PAGE Analysis of Bacterial Proteins

SDS-PAGE was performed to analyze the dialyzed protein fractions. The electrophoretic mobility of the proteins was mainly according to their molecular weight and molecular size. The SDS–PAGE apparatus was washed thoroughly with double distilled water. Glass plates were sandwiched by the spacers and were tightly clamped. Approximately 12% separating gel or resolving gel was prepared and poured and was allowed to polymerize, on which the separating gel buffer was poured to prevent the drying of the gel. Once the separating gel was set, the buffer was removed, and the gel was washed with tank buffer. The freshly prepared 4% stacking gel was poured on the top of the separating gel, and the comb was inserted and was left undisturbed to polymerize. The gel was covered by the stacking gel buffer. Once the gel was set, the comb was removed carefully, and the wells were washed with a tank buffer to remove unpolymerized acrylamide. The dialyzed sample was treated with 1X sample lysing buffer, consisting of 0.1 M Tris HCL (pH 6.8), 1% SDS, 10% glycerol, 5% β-mercaptoethanol and 0.001% Bromophenol blue. Approximately 20 µL of the sample was mixed with an equal volume of the sample loading buffer and heated at 95 °C for 5 min in a boiling water bath. The polymerized gel was placed between the two buffer reservoirs where the notched plate faced towards the buffer reservoir. The gel was covered by 1X tank buffer, and approximately 20 µL of the sample was loaded into each well. The upper reservoir had a negative electrode (cathode), and the lower had a positive electrode (anode). Power was applied where initially 50 V was applied, and once the sample reached the separating gel, the voltage was increased to 100 V. Power was switched off when the bands migrated towards the bottom, and the gel was removed. Followed by electrophoresis, the gels were stained by staining solution, and subsequently, the gels were destained overnight. The bands were viewed under a UV transilluminator [34].

### 3.7. Synergism Test by Checkerboard Method

The checkerboard broth macro dilution method was used to determine the synergistic activity between the antibiotic, streptomycin sulphate and methanolic extract of *S. glauca*. Two-fold serial dilutions of the antibiotic and plant extracts were prepared in the concentration of 200 µg/mL to 0.1 µg/mL, respectively. Approximately 100 µL of *Staphylococcus aureus* (1.5 × 10^8^ CFU/mL) was inoculated and incubated at 37 °C for 24 h. The synergism was assessed with the help of the fractional inhibitory concentration index (FICI) [35]. The FICI was calculated as:

FIC C=FIC A+FIC B

$$FIC\;A=\frac{MIC\;of\;plant\;extract\;in\;combination}{MIC\;of\;plant\;extract\;\left(A\right)}$$

$$FIC\;B=\frac{MIC\;of\;streptomycin\;incombination}{MIC\;of\;streptomycin\;\left(B\right)}$$

The FIC index was interpreted as:

(i) Synergistic effect if FIC < 0.5
(ii) Additive/indifference if 0.5 < FIC < 4
(iii) Antagonist effect if FIC > 4

### 3.8. Isolation of Bioactive Compound through Column Chromatography

The bioactive compound of the crude methanolic extract of *S. glauca* was isolated according to the procedure of Khan et al. [36]. Briefly, the glass column was rinsed with distilled water followed by methanol and was allowed to dry. An adsorbent silica gel G of 60–120 mesh size acted as a stationary phase. The extract was allowed to run in the column. The eluted fractions were then subjected to antibacterial activity against *E. coli*, *S. aureus* and *S. marcescens* to determine the efficacy of bioactive compounds.

### 3.9. Identification of Bioactive Compound

#### 3.9.1. Fourier Transform Infra-Red (FTIR) Analysis of Column Fraction

The bioactive compound of the plant extract was identified using FTIR equipped with a DLATGS detector with temperature control [21]. Briefly, the column fraction that exhibited prominent antibacterial activity was analyzed for its functional group. Approximately 10 mg of dried extract powder was encapsulated in 100 mg of KBr pellet in order to prepare a translucent sample disc. The sample was then loaded in FTIR spectroscopy (Shimadzu, IR Affinity1, Kyoto, Japan) with a scan range of 4000 to 400 cm^−1^ and resolution of 0.9 cm^−1^.

#### 3.9.2. Gas Chromatography-Mass Spectroscopy (GC-MS)

The fraction that was separated through the column was identified through GC-MS according to the procedure of Kalaisezhiyen et al. [37]. The proactive bioactive compounds present in the methanolic extract of *S. glauca* were identified using GC-MS (Shimadzu QP 2010 plus, Tokyo, Japan) equipped with a silica column (5 MS-RT × 1). Pure helium gas (99.99%) was used as the carrier gas at a constant flow rate of 1 mL/min. For GC–MS spectral detection, the mass range was set as *m*/*z* 1.5 to 1000, and an electron ionization (EI) mode was adopted. The EI scan sensitivity was set to 1 pg octafluoronaphthalene *m*/*z* 272 S/N > 200. A turbomolecular pump with 58 L/sec for He and a Rotary pump with 30 L/min (60 Hz) was used. The contents of phytochemicals present in the *S. glauca* extract were identified based on a comparison of their retention time (min), peak area, peak height and mass spectral patterns with the standard compounds available in the National Institute of Standards and Technology (NIST) library in order to detect the name and structure of the compounds.

#### 3.9.3. Analytical Thin Layer Chromatography (TLC) of Column Fraction

TLC was performed according to the method followed by Connolly et al. [38] and Rajiniraja et al. [23]. The methanolic extract of the samples was spotted using a thin capillary tube. The solvent front was allowed to run. Then, the TLC plate was removed and air-dried. It is then sprayed with 2 mg/mL of 2, 3, 5 triphenyl tetrazolium chloride, dried and is visualized under UV light. The chromatograms were marked, and retention factor (*Rf*) values were calculated by using the formula,

$$Retention\;factor\;\left(Rf\right)=\frac{Distance\;travelled\;by\;the\;solute\;\left(plant\;extract\right)}{Distance\;travelled\;by\;the\;solvent}$$

Followed by which the bioactive property of the separated compound was determined.

### 3.10. Antioxidant Activity of S. glauca Extract

The free radical scavenging activity of *S. glauca* extracts was assessed with a DPPH assay. Briefly, the concentrations, 50 µg/mL, 500 µg/mL and 1000 µg/mL of extracts were prepared in methanol and ethanol, respectively. To 1 mL of each solution, 2.5 mL of 0.2 M phosphate buffer (pH 6.6), 2.5 mL of 1% potassium ferricyanide was added and mixed. It was then incubated at 50 °C for 20 min. It was then centrifuged at 3000 rpm for 10 min, and 2.5 mL of 10% trichloroacetic acid was added to the mixtures. The upper layer or supernatant (5 mL) was mixed with an equal volume of distilled water and 1 mL of 0.1% ferric chloride. The intensity of the color developed was directly proportional to the reducing power of the antioxidants present. Ascorbic acid of a similar concentration was prepared and used as standard. The absorbance of both the sample and standard was measured at 700 nm [39].

### 3.11. Anticancer Activity of S. glauca Extract by MTT Assay

The extract that exhibited higher antioxidant activity was further analyzed for its cytotoxic or anticancer activity. The human breast cancer cell lines (MCF 7) were obtained from the National Centre for Cell Science (NCCS), Pune, India. The cells were subcultured in eagle’s minimum essential medium supplemented with 10% fetal bovine serum (FBS). The cells were maintained at 37 °C, 5% CO_2_, 95% O_2_ and 100% relative humidity. The monolayer cells are separated with trypsin- ethylenediaminetetraacetic acid (EDTA) to make single-cell suspensions. The cell viability was tested by trypan blue exclusion method using a haemocytometer. A known number of cells 1 × 10^5^ cells/well in 100 µL medium were seeded into 96 well plates, respectively, for performing the MTT assay. The samples were initially dissolved in dimethylsulfoxide (DMSO). The cells were treated with different concentrations of the plant extract such as 6.25 µg/mL, 12.5 µg/mL, 25 µg/mL, 50 µg/mL and 100 µg/mL, respectively, and incubated for 48 h at 37 °C with 5% CO_2_, 95% O_2_ and 100% relative humidity. After 48 h of incubation, approximately 15 µL of MTT (3-[4,5-dimethylthiazol-2-yl]2,5-diphenyltetrazolium bromide) was added to each well and incubated at 37 °C for 4 h. After incubation, the mixture was solubilized in 100 µL of DMSO. The absorbance of suspension was recorded at 570 nm using a microplate reader. The percentage (%) cell inhibition was calculated by using the following formula,

$$\%\mathrm{Cell}\;\mathrm{Inhibtion}=100-\frac{Absorbance\;of\;sample}{Absorbance\;of\;control}\times 100$$

The IC_50_ was determined using GraphPad Prism 9 software [25,40].

## 4. Conclusions

The overall results of our study prove that the methanol extract of *S. glauca* has profound antibacterial activity on the multi-drug resistant strain *Staphylococcus aureus*. It is also further known to imbibe predominant antioxidant and anticancer activity. The plant extract proves to have promising anticancer potential by inhibiting the MCF-7 breast cancer cell line. However, the search for new lead compounds from natural sources with more effectivity and less toxicity would constitute an interesting alternative for the development of anticancer drugs in breast cancer treatment. Thus, further studies are needed to evaluate the potential lead fraction of the active plant and its mechanism of action. This will help us take further steps in herbal based drug development which would relieve human ailments.

## Figures and Tables

**Table 1:** Phytochemical screening of leaf extract of *Simarouba glauca*.

Phytochemicals	Test Performed	Methanol	Ethanol
Alkaloids	Mayer’s test	Absent	Absent
	Hager’s test	Present	Present
	Wagner’s test	Present	Present
Flavonoids	Alkaline Reagent test	Present	Present
	Ferric Chloride test	Present	Present
Phenolic compounds	Ferric Chloride test	Present	Present
Steroids and Terpenoids	Liebermann–Burchard test	Present	Present
Tannins	Ferric Chloride test	Present	Present
Glycosides	Keller kiliani test	Present	Present
Saponins	Foam test	Present	Present
Carbohydrates	Molisch’s test	Present	Present
Proteins	Biuret test	Absent	Absent
Aminoacids	Ninhydrin test	Absent	Absent
Fixed oils	Spot test	Present	Present

**Table 2:** Antimicrobial activity of plant extracts.

Microbial Pathogens	Zone of Inhibition (mm)		
	Methanol (100 mg/mL)	Ethanol (100 mg/mL)	Streptomycin
**Bacterial Pathogens**			
*Acinetobacter baumannii*	-	-	-
*Escherichia coli*	20.00	19.00	18.00
*Klebsiella oxytoca*	-	-	13.00
*Klebsiella pneumoniae*	-	-	19.00
*Proteus mirabilis*	18.00	18.00	20.00
*Proteus vulgaris*	-	-	20.00
*Salmonella typhi*	18.00	17.00	20.00
*Serratia marcescens*	20.00	19.00	18.00
*Shigella sonnei*	18.00	15.00	25.00
*Bacillus* sp.	16.00	16.00	20.50
*Enterococcus faecalis*	12.00	12.00	-
MRSA	21.00	17.00	20.00
*Staphylococcus aureus*	24.00	23.00	19.00
*Micrococcus luteus*	17.00	15.00	16.00
**Fungal pathogens**			**Fluconazole**
*Aspergillus fumigatus*	14.00	13.00	15.00
*Aspergillus niger*	18.00	14.00	16.00
*Candida albicans*	-	10.00	19.00
*Mucor* sp.	12.50	-	-
*Penicillium* sp.	10.00	13.00	14.00
*Rhizopus* sp.	14.00	13.50	-

**Table 3:** Breakpoints of *S. glauca* extract.

Test Organism	Methanol (100 mg/mL)	Ethanol (100 mg/mL)	Streptomycin (100 mg/mL)
*E. coli*	6.30	12.50	6.30
*P. mirabilis*	25.00	25.00	12.50
*S. typhi*	6.30	6.30	6.30
*S. marcescens*	6.30	12.50	6.30
*S. sonnei*	12.50	6.30	3.20
*Bacillus* sp.	3.20	12.50	1.60
*E. faecalis*	25.00	12.50	-
MRSA	6.30	6.30	3.20
*M. luteus*	6.30	1.60	25.00
*S. aureus*	3.20	6.30	1.60

**Table 4:** FIC values and FIC index of antibiotic and plant extract.

MIC Combination	Breakpoints ^$^		FIC A	FIC B	FIC Index
½ MIC of extract	1.6		0.125	2.00	2.25 (Indifference)
½ MIC of Streptomycin	0.8				
½ MIC of extract + ½ MIC of Streptomycin	3.2 (A)	1.6 (B)			
MIC of plant extract	3.2		0.25	0.25	0.50 (Synergistic)
MIC of Streptomycin	1.6				
MIC of extract + MIC of Streptomycin	0.8 (A)				
2× MIC of extract	1.6		0.25	0.125	0.375 (Synergistic)
2× MIC of Streptomycin	1.6				
2× MIC of extract + 2× MIC of Streptomycin	0.4 (A)	0.2 (B)			

^$^ Breakpoints of extract in mg/mL; Breakpoints of streptomycin in µg/mL; **A**: Plant extract; **B**: Streptomycin.

**Table 5:** Separated column fractions.

Column Fractions	Color	Zone of Inhibition (in mm)		
		*S. aureus*	*E. coli*	*S. marcescens*
F1	Dark green	12.00	13.50	11.00
F2	Light green	16.00	14.00	10.70
F3	Brown	21.00	16.50	11.50
F4	Pale green	13.00	10.00	9.50

**Table 6:** FTIR absorbance values of the F3 column fraction.

Absorption Peak (cm^−1^)	Bond	Functional Groups
3425.58	O-H strech	Carboxylic acids
2924.09, 2854.65, 2376.3	C-H strech	Alkanes
1705.07–1720.8	C=O stretch	Ketone
1627.92	N-H bend	Amines
1450.47, 1373.32	C-H strech	Alkanes
1327.03	C-N stretch	Aromatic amines
1249.87	C-N stretch	Aromatic amines
1056.99–671.23	C=C bend	Aromatic compounds

**Table 7:** GCMS analysis of F3 column fraction of *S. glauca.*

Peak No	Retention Time	% Peak Area A/H	Compound Name	Compound Nature	Structure
1	5.705	2.61	Cyclohexanone	Terpenoid, oil with ketone group	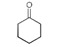
2	16.757	2.3	Benzoic acid, 2.6-bis (Trimethylsiloxy)	Aromatic carboxylic acid	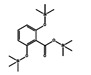
3	19.926	1.63	Squalene, 2,6,10,14,18,22-Tetracosahexaene	Trierpenoid	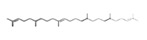
4	21.883	2.26	Cyclopentanol	Cyclic Alcohol	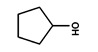
5	22.715	2.92	Pthalic acid, Cyclobutyl isobutyl ester	Aromatic dicarboxylic acid	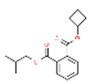
6	24.179	3.03	3-ethyl-3-methyl Decane	Alkane hydrocarbon	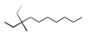
7	26.266	3.31	(2,4,4,6,6,8,8-Heptamethyltetrasiloxan-2-yloxy)	Alkene	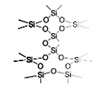
8	34.601	5.17	7-Oxabicyclo(4.1.0)Heptane	Alkane	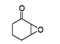

**Table 8:** Anticancer activity of *Simarouba glauca* extract.

Concentration of Extract (µg)	Absorbance at 570 nm	% Cell Inhibition ^$^
6.25	0.398	18.10
12.50	0.288	40.70
25.00	0.150	67.14
50.00	0.070	84.01
100.00	0.030	93.41
Control	0.486	-

^$^ values are mean inhibition ± S.D of three replicates.

## Data Availability

The data presented in this study are available on request from the corresponding author.
